# Sleep Circulation Time from Pulse Oximetry and Polysomnography: Predictive Value in Patients with Heart Failure with Reduced Ejection Fraction

**DOI:** 10.3390/s26154849

**Published:** 2026-08-01

**Authors:** Wei Jung Hsia, Jack Rodman, Benjamin Cantrill, Richard J. Castriotta

**Affiliations:** 1 Division of Pulmonary, Critical Care and Sleep Medicine, Keck School of Medicine, University of Southern California, Los Angeles, CA 90023, USA; weijung.hsia@med.usc.edu (W.J.H.); cantrill.ben@gmail.com (B.C.); 2 Southern California Clinical and Translational Science Institute, University of Southern California, Los Angeles, CA 90089, USA; jrodman@usc.edu

**Keywords:** sleep apnea, apnea-hypopnea index, circulation time, ejection fraction, heart failure, pulse oximetry, polysomnography, cardiovascular disease, sleep-disordered breathing

## Abstract

**Highlights:**

**What are the main findings?**

- Circulation time can be estimated from pulse oximetry during polysomnography (PSG) from the pulse oximetry-measured recovery time of oxygen saturation after an apnea or hypopnea event.
- All subjects with left ventricular ejection fraction ≤45% had prolonged circulation time (median of 27.8 s).

**What are the implications of the main findings?**

- Circulation time may be used to identify the presence of heart failure in polysomnography.
- Those with sleep circulation time over 28.6 s on PSG should have an evaluation of cardiac function.

**Abstract:**

*Background*: This study evaluated the use of circulation time (Tcirc) calculated from polysomnogram (PSG) with pulse oximetry to identify poor cardiac function with low left ventricular ejection fraction (EF). *Methods*: Subjects over 18 years of age with sleep apnea (apnea-hypopnea index (AHI) > 5/h diagnosed by PSG who had transthoracic echocardiography (TTE) within 1 year of PSG were included in this retrospective study. Tcirc of each sleep stage (N2, N3, and REM) were measured and averaged and EF was recorded. Statistical analysis was carried out using the Wilcoxon rank sum test, logistic regression and Youden index. *Results*: There were 89 subjects who met the inclusion criteria, 14 with EF ≤ 45% (Group A) and 75 with EF ≥ 50% (Group B). The normal Tcirc is <20 s, but Group A subjects had a prolonged overall Tcirc with a median time of 27.8 s (range 14.1–39.6 s), compared to Group B subjects with a median Tcirc of 23.5 s (range 14.3–37.6 s), *p* = 0.311. The optimal cut-point for overall sleep Tcirc with moderate discrimination (AUC = 0.6) was 28.6 s. Those with total sleep Tcirc ≥ 28.6 s were 2.5× more likely to have low EF with OR = 2.56 (95% CI, 0.55–11.16). *Conclusions*: In sleep apnea patients, total sleep Tcirc > 28.6 s is associated with low ejection fraction with specificity = 0.78.

## 1. Introduction

Non-invasive transcutaneous pulse oximetry has long been utilized to estimate arterial oxygen saturation [1], facilitating the revelation of sleep-related hypoxemia in chronic obstructive pulmonary disease (COPD) [2,3,4] and sustained sleep hypoxemia-induced right ventricular dysfunction in COPD patients [5]. The intermittent sleep hypoxemia seen in obstructive sleep apnea (OSA) [6] leads to systemic sleep hypertension, left ventricular dysfunction and increased cardiovascular events. Recently this measurement of nocturnal oxygen saturation via pulse oximetry has led to the concept of hypoxic burden [7] and the revelation of its correlation with cardiovascular events [8] and mortality [9] in OSA. Azarbarzin et al. found an association between hypoxic burden and cardiovascular disease and all-cause mortality in men aged 65 years or older [10], but no correlation with AHI. Oldenburg et al. showed that hypoxic burden (defined as time spent with oxygen saturation <90%) is the strongest independent predictor for all-cause mortality in HF patients. They used a cutoff point of 22 min to best predict mortality [11].

We now realize that pulse oximetry in sleep apnea subjects during polysomnography (PSG) can be utilized to estimate not only hypoxic burden, but also the length of time for recovery of oxygen saturation after cessation of apnea and resumption of breathing: the circulation time (Tcirc), which is normally <20 s.

The concept of sleep circulation time (Tcirc) has been described in the past. Tcirc is also known as the lung to periphery time. Hall et al. [12] hypothesized that lung-to-carotid body circulatory delay may explain the cycle length of periodic breathing, which is reflected by lung-to-finger or lung-to-ear circulation time, otherwise known as the lung to periphery time. This can be identified on the PSG as the time interval from the start of hyperpnea following an apnea or hypopnea, to the lowest point (i.e., nadir) of oxygen saturation measured at a peripheral site by pulse-oximetry [13]. A prolonged Tcirc is a hallmark of heart failure with increased mortality risk, and a non-invasive method of detecting prolonged Tcirc during PSG might lead to detection of undiagnosed heart disease. This would also identify the cause of any observed central sleep apnea (CSA) or Cheyne-Stokes respiration (CSR).

Presence of Sleep-disordered breathing (SDB) has been strongly associated with heart failure (HF) [14]. Obstructive sleep apnea (OSA), CSA and CSR are associated with increased mortality in those with HF [15,16]. Therefore, identifying markers of heart failure in PSGs may aid the early identification and management of heart disease, with the potential benefit of reduced mortality and morbidity.

Prior studies have utilized Tcirc as a marker for heart failure and mortality. It was shown that prolonged sleep Tcirc may be associated with worse outcome [17,18]. Hall et al. demonstrated that Tcirc is prolonged in those patients with CSR associated with HF compared to idiopathic CSA. They also demonstrated that the lung to ear circulation time is inversely related to cardiac output [12]. Therefore, it is thought that prolonged Tcirc is an indicator for low cardiac output state. Kwon et al. found that the most significant factor associated with delayed lung to finger circulation time (LFCT) was the presence of HF, and prolonged LFCT was associated with greater CV and all-cause mortality [18].

Bitter et al. used cycle length in the diagnosis of central and obstructive sleep apnea (CSA/OSA) [19]. Circulatory delay (CD) was measured the same way as Tcirc. They found that in patients with heart failure with reduced left ventricular ejection fraction (HFrEF), Tcirc was prolonged.

In our study, we sought to use Tcirc as a marker for heart failure and to identify cut off points which may reliably indicate the presence of HFrEF.

## 2. Methods

### 2.1. Subjects

We included subjects who are 18 years or older, with sleep apnea, defined as apnea-hypopnea index (AHI) > 5/h, diagnosed by PSG who also had transthoracic echocardiography (TTE) within 1 year of PSG. We excluded people with known diastolic dysfunction (*n* = 38) and borderline EF = 40–49% (n = 13). All patients meeting these inclusion/exclusion criteria were used in the study. Subjects’ demographic information including gender, age, ethnicity, BMI, and beta blocker usage were obtained from electronic medical records taken from Keck Hospital of USC in Los Angeles, California, between January 2021 and March 2023. TTE was reviewed by a Board-certified cardiologist and EF and diastolic function were recorded. PSGs for each patient were reviewed by a Board-certified sleep medicine specialist, AHI and hypoxic time were obtained as well as circulation time (see below for circulation time measurements). Hypoxic time was determined by time spent with an oxygen saturation (SpO_2)_ below 88% (T88) recorded by pulse oximetry during the PSG. This study was approved by Institutional Board Review at University of Southern California (HS-20-00769).

### 2.2. Circulation Time Measurements

Polysomnography was performed using Natus XLTEC Sleepworks version 9.6. This Natus polysomnography system (Natus Medical Inc., Middleton, WI, USA) is a state-of-the-art standard used in many sleep disorder centers accredited by the American Academy of Sleep Medicine. Tcirc was measured during PSG from the start of hyperpnea following the end of an apnea or hypopnea, to the SpO_2_ nadir measured by a Nonin pulse-oximeter with model 6000CA-WO2 sensor on the finger (Nonin Medical Inc., Plymouth, MN, USA). The Nonin pulse oximeter with PureSAT technology has been validated with precision of ±2.1 (https://www.nonin.com/resource/accuracy-and-superior-performance-of-puresat-and-purelight-oximetry-technologies/, accessed 27 July 2026). Three Tcirc values were measured from the first, middle, and last third of each sleep cycle (stages N2, N3, and REM), and we then averaged nine Tcirc measurements from each of stages N2, N3, and REM stages as the average Tcirc. Total sleep Tcirc was defined as the combined average Tcirc between N2 + N3 + REM. All Tcirc measurements were done by the same investigator.

### 2.3. Statistical Analysis

Summary statistics were presented using frequency and percent for categorical variables and mean (SD) or median (IQR) for continuous variables, dependent on distribution. Independent samples *t*-test or Wilcoxon rank sum test, as appropriate, was used to evaluate differences in Tcirc time between subjects with EF ≥ 50% and EF ≤ 45%. The choice to use a Wilcoxon rank sum test was based on the skewness of the variable being tested as well as the small and unequal group sizes, such as the small EF group. Optimal cut-points for REM Tcirc average and total sleep Tcirc average were evaluated based on Youden’s Index (criterion for maximizing sum of sensitivity and specificity). Diagnostic measures of accuracy (AUC/ROC curve, sensitivity, specificity) were used for interpretation and comparison of cut-points. Multivariable firth’s logistic was used to evaluate the association between identified REM groups and likelihood of low EF (≤45%), while controlling for age, BMI, and patient beta blocker status. All tests were two-sided and a *p*-value < 0.05 was considered statistically significant. All analyses were done in R version 4.2.3.

## 3. Results

A total of 89 subjects were included: 14 subjects with EF ≤45% (Group A), 75 subjects with EF ≥ 50% (Group B). Patient characteristics are listed in Table 1.

### 3.1. Circulation Time

**Figure 1:**
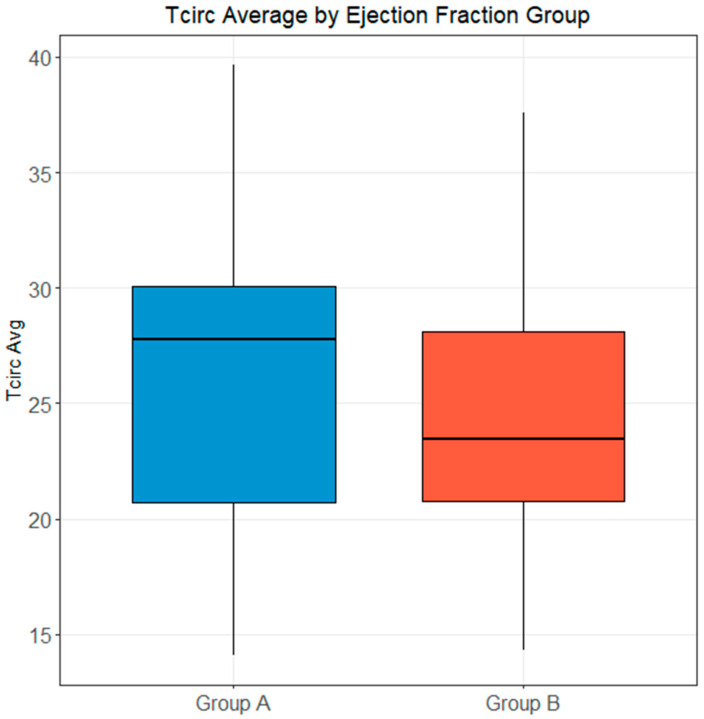
Tcirc distribution between EF groups.

- All 14 Group A subjects (with low EF) had prolonged overall Tcirc with a median time of 27.8 s (normal <20 s) and interquartile range (IQR) of 3.9, compared to Group B subjects with median Tcirc of 23.5 s with IQR = 7.3 (Figure 1; Table 2). The Tcirc ranges for these groups were 14.3–37.6 s and 14.1–39.3 s respectively. There were 2 subjects in Group A and 6 subjects in Group B who had no REM sleep on PSG. Of those subjects who did exhibit REM sleep, all in Group A had prolonged Tcirc (median 29 s, range 21–36.5 s) compared to Group B (25.5 s range 16.1–51.2), *p* = 0.048. Because ejection fraction was measured as an ordinal variable, a Kendall’s Tau-b (τ-b) correlation was used. This showed a moderately weak, negative, but nonsignificant, relationship between Tcirc and ejection fraction, *r_Ƭ__b_* = −0.11, *p* = 0.278. During REM sleep, those in Group A had a significantly higher circulation time (median 35 s, range 26.3–39) compared to Group B (25.5 s, range 16.1–51.2) *p* = 0.048 (Table 2).

### 3.2. Optimal Cut Points for Circulation Time

**Figure 2:**
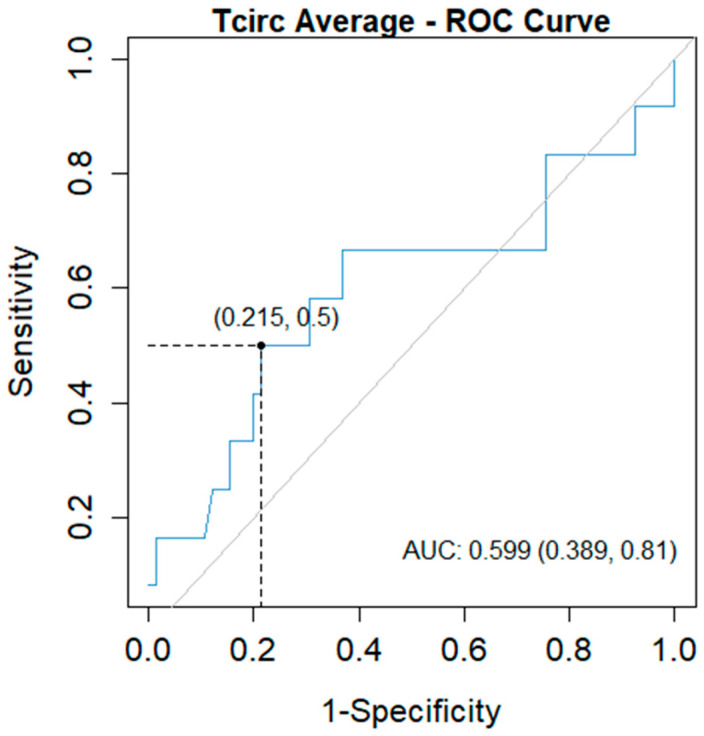
Receiver Operator Curve (ROC) for Tcirc average cut-point analysis.

**Figure 3:**
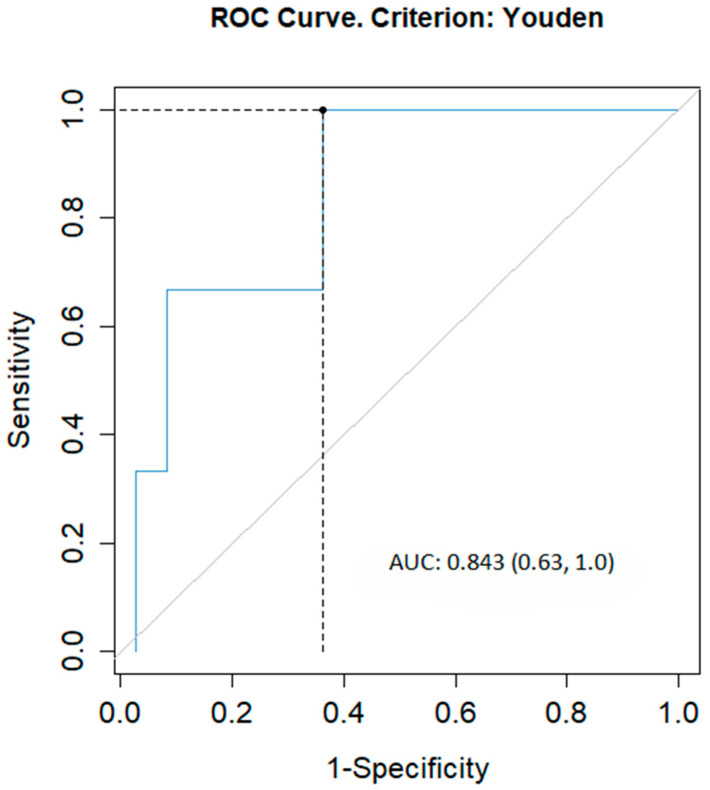
Receiver Operator Curve (ROC) for Tcirc average in REM sleep.

The optimal cut-point for total sleep Tcirc average with moderate discrimination (AUC = 0.60. CI 0.39–0.81) was 28.6 s, with sensitivity = 0.5 and specificity = 0.78 (Figure 2; Table 3). Those with sleep Tcirc ≥ 28.6 s were 2.5 times more likely to have low EF (OR = 2.56; 95% CI = 0.55–11.16). The optimal cut-point for REM sleep was 26.3 s with excellent discrimination (AUC = 0.84, CI 0.63–1.0), sensitivity = 1.0, specificity = 0.64 (Figure 3). Those with REM Tcirc ≥ 26.3 s were 12 times more likely to have low EF (OR = 12.2, CI 0.54–272.5).

### 3.3. Beta Blocker Use and Circulation Time

Using the calculated optimal cut points for circulation time of 28.6 s, it was found that patients who are not on beta blockers were less likely to have prolonged Tcirc (OR = 0.84; 95% CI 0.03–26.70), whereas those with beta blocker use were more likely to have prolonged Tcirc ≥ 28.6 s (OR= 1.30; 95% CI = 0.19–8.86), Table 4.

Since the observed significant difference in Tcirc during REM sleep could be related to beta-blocker use, we evaluated the subjects on beta blockers separately, reducing Group A to 10 and Group B to 25 subjects (Table 5). This showed no significant difference in Tcirc during REM sleep among those taking beta blockers, indicating that the significant difference Tcirc among the total group is not due to beta blocker use. It may be that the wide autonomic nervous system fluctuations during REM sleep are attenuated with beta blockers, and that the detrimental effects of REM sleep on cardiac function are more manifest. Normally our lowest cardiac output of the 24 h day occurs during the last REM cycle.

### 3.4. AHI and Hypoxic Time

The subjects in Group A had a median AHI of 40.8 (range 6.8–109) with IQR = 30.4 compared to those in Group B (AHI = 20.7 (range 5–136), IQR = 17.7, *p* = 0.247), Table 6. Group A subjects had a median hypoxic time < 88% (T88) of 9.55 min (3.8–30.1 min), IQR = 17.2, versus those in Group B with median of 6.1 min (range 0.1–159 min), IQR = 23.8, Table 7.

## 4. Discussion

### 4.1. AHI and Hypoxic Burden in Heart Failure with Reduced Ejection Fraction

Over the last few decades, the data on association between AHI and HF and cardiovascular mortality has been conflicting. Punjabi et al. showed correlation of AHI with all-cause mortality and coronary artery disease in men aged 40–70 years old, especially in those with AHI > 30/h, but not with women or with men > 70 years [20]. The data correlating AHI and HFrEF is sparse. A prospective study done by Gottlieb et al. [21] demonstrated AHI to be associated with HF in men. They showed that men with AHI ≥ 30/hr were 58% more likely to develop HF in comparison to those with AHI < 5/hr. Azarbarzin et al. did not find significance in AHI and incidence of HF [22]. In our study, the AHI was higher in those with EF ≤ 45% compared to those with normal EF (40.8 vs 20.7). More importantly, it has been shown that sleep hypoxic burden predicts incidence of HF in men [18]. Yuksel et al. found an association between hypoxic burden and major cardiovascular and cerebrovascular events in sleep apnea patients [23]. Additionally, Khouzani et al. showed that oxygen saturation < 85% was a better predictor of mortality than AHI in OSA [8]. In our study, there was a trend of longer hypoxic time in those with EF ≤ 45% vs those with normal ejection fraction, but this did not reach statistical significance. However, hypoxic time in our study was determined by oxygen saturation < 88%. It is likely that AHI and hypoxic time with oxygen saturation < 88% do not capture the pathology of true hypoxic burden effecting heart failure. While apnea index > 20 apneas/hour may indicate increased mortality [24], the value of AHI is diluted by hypopneas with little or no hypoxemia. Therefore, using other markers such as Tcirc that correlate with hypoxic burden may serve to identify those with HF and higher mortality risk.

### 4.2. Circulation Time and Cardiac Output

In 2015, Hosokawa et al. [25] demonstrated cardiac output and cardiac index measured by right heart catheterization significantly correlate with the LFCT measured by overnight PSG. We confirmed this in our study in which Tcirc during sleep is longer in patients who have EF ≤ 45%. All subjects with EF ≤ 45% had prolonged circulation time (median of 27.8 s vs. normal < 20 s). This suggests that Tcirc may be used to identify the presence of heart failure, and perhaps Tcirc should be reported as part of the PSG measurements. It is further confirmed in multivariable analysis as the use of beta blockers is generally associated with presence of heart failure, and those who are taking beta blockers are more likely to have prolonged Tcirc. In our study, the REM sleep Tcirc was significantly higher in those with low EF (*p* = 0.48) and was the best predictor of prolonged EF with median Tcirc = 35 s and OR = 12.2. This may be due to the distinct physiology of REM sleep with fluctuations in coronary artery blood flow, changes in systemic blood pressure up to 40 mmHg, the lowest cardiac output over 24 h span, and elevated risk of coronary artery spasms [19]. The difficulty in accurately measuring the REM Tcirc specifically renders it difficult for clinical practice. This may be less of a problem in the future with the implementation of artificial intelligence as a tool in polysomnography.

### 4.3. Circulation Time Predicting HFrEF

Wang et al. [12] published their finding that in patients with HF, untreated OSA is independently associated with higher mortality. Recently, Bitter et al. [20] used cycle length in the study of central and obstructive sleep apnea (CSA/OSA). They studied heart failure patients using cycle length in central sleep apnea, while we studied all sleep apnea patients and found a subset who had heart failure. Circulatory delay (CD) was measured the same way as Tcirc in our study, and they found that in HFrEF patients, the optimal CD cut off was 26.4 s with AUC = 0.79. This may be compared to our optimal cut-off point of 28.6 s (AUC = 0.60) with OR = 2.56 for low EF ≤ 45%. This suggests that patients with high circulation time above the optimal cut-off point should have an echocardiogram to evaluate the possible presence of HF with low EF.

### 4.4. Study Limitations

Our study has several limitations. First, this is a retrospective study. We did not exclude subjects with BMI > 45 kg/m^2^, and morbidly obese subjects may constitute a distinct type of hypopnea-dominant SDB and may have concomitant obesity hypoventilation syndrome and/or a distinct hypopnea-dominant OSA [26]. We also noted a wide range of Tcirc amongst the patients, which may affect the data. This might be explained by various degrees of severity in patient’s HF for those EF ≤ 45%. We chose median instead of mean data to account for the degree of variation in Tcirc. In addition, EF can be dependent on the echocardiographer’s interpretation. To avoid such bias, we choose EF ≥ 50% and EF ≤ 45% to avoid the discrepancies. Further, the use of beta blockers could bias the study, as HF patients tend to be on beta blockers which has been shown to be associated with prolonged circulation time [27]. We did not exclude smokers or those with elevated carboxyhemoglobin, which may reflect the accuracy of pulse oximetry [28], and we did not take note of skin color or pigmentation, which may also influence SpO_2_ estimates by pulse oximetery [29,30]. The multivariable statistical models did not adjust for many potential confounders (hypertension, diabetes, chronic kidney disease, coronary artery disease, smoking status). This should be corrected in future studies. Despite our limitation, we believe that Tcirc during PSG may be a good indicator of cardiac function. The wide confidence interval is mostly a result of the very small sample size in the analysis and the fact that this study was relatively underpowered. The small sample size may explain the failure to reach statistical significance, but nevertheless, these results may be of clinical significance. This may be viewed as an exploratory study primarily examining the predictability of Tcirc in total sleep and REM sleep. Certainly, more controlled and larger studies are needed to confirm these results and determine their statistical significance.

### 4.5. Future Direction

A future prospective study with PSG and pulse oximetry and TTE days apart can provide the most accurate results for optimal cut off points for predicting HFrEF. We can also further stratify patients between OSA and CSA to see if there are additional differences. It is also reasonable to suspect that the Tcirc estimated by our method should also be valid using a home sleep apnea test (HSAT) rather than in-lab PSG. Future evaluation of Tcirc via HSAT will be forthcoming in view of the increasing utilization of HSATs in the medical community.

## 5. Conclusions

Our findings suggest that those with low EF have prolonged sleep circulation time, which can be estimated during PSG with pulse oximetry. Our analysis suggests that those with sleep Tcirc > 28.6 s or REM sleep Tcirc > 26.3 s on PSG should have an evaluation of cardiac function.

## Figures and Tables

**Table 1:** Demographics.

Variable	EF Group		*p*-Value
	Group B (EF ≥ 50%) (n = 75)	Group A (EF ≤ 45%) (n = 14)	
Median Age (years)	64.0 (min = 23, max = 94)	67.5 (min = 25, max = 83)	0.414
Gender			0.333
Male	52 (69.3%)	12 (85.7%)	
Female	23 (30.7%)	2 (14.3%)	
Ethnicity			0.004 *
White/Caucasian	32 (45.7%)	2 (16.7%)	
Black/AA	1 (1.4%)	2 (16.7%)	
Asian	5 (7.1%)	4 (33.3%)	
Hispanic	29 (41.4%)	3 (25.0%)	
Other	3 (4.3%)	1 (8.3%)	
Median BMI (kg/m^2^)	30.1 (9.4) (min = 17.3, max = 56.0)	30.6 (9.7) (min = 22.5, max = 47.1)	0.978
Type of Sleep Apnea			0.009 *
Central	5 (6.7%)	2 (14.3%)	
Obstructive	66 (88.0%)	8 (57.1%)	
Complex	4 (5.3%)	4 (28.6%)	
Beta Blocker Use			0.007 *
No	49 (67.1%)	4 (28.6%)	
Yes	24 (32.9%)	10 (71.4%)	

* Indicates statistical significance.

**Table 2:** Median sleep circulation time (seconds).

Variable	EF Group		*p*-Value
	Group B (EF ≥ 50%)	Group A (EF ≤ 45%)	
Total Sleep Tcirc (Range)	23.5 14.3–37.6	27.8 14.1–39.3	0.311
REM Sleep Tcirc	35 26.3–39	25.5 16.1–51.2	0.048

**Table 3:** Optimal Cut-Point Analysis for Prediction of Low EF.

Variable	Optimal Cut-Point Value	AUC (95% CI)	Sensitivity	Specificity
N2 + N3 + REM Sleep	28.6 s	0.60 (0.39–0.81)	0.50	0.78
REM Sleep	26.3 s	0.84 (0.63–1.0)	1.0	0.64

AUC = Area Under Receiver Operator Characteristic (ROC) Curve.

**Table 4:** Multivariable logistic regression of patients with and without beta blockers (using Tcirc > 28.6 s).

	Odds Ratio (95% CI)	*p*-Value
Without Beta blocker use	0.84 (0.03–26.70)	0.919
With Beta blocker use	1.30 (0.19–8.86)	0.790

**Table 5:** Median sleep circulation time (seconds). Beta Blockers-only group.

Variable	EF Group		*p*-Value
	Group B (EF ≥ 50%)	Group A (EF ≤ 45%)	
Total Sleep Tcirc (Range)	23.6 16.8–37.6	27.2 14.1–39.3	0.704
REM Sleep Tcirc	21.6 18.5–33.9	19.6 15.4–38.2	0.598

**Table 6:** AHI by EF level.

Variable	EF Group		*p*-Value
	Group A (EF ≤ 45%) (n = 14)	Group B (EF ≥ 50%) (n = 76)	
Median AHI (events/hour)	40.8 Range 6.8–109.0	20.7 Range 5.0–136.0	0.247

**Table 7:** Hypoxic time (minutes) and percent desaturation by EF Group.

Variable	EF Group		*p*-Value
	Low EF (≤45%) (n = 14)	Normal EF (≥50%) (n = 76)	
Median Hypoxic Time (minutes)	9.55 Range 3.8–30.1	6.10 Range 0.1–159.0	0.378

## Data Availability

Data from this project will be made available upon request to the corresponding author.

## Abbreviations

The following abbreviations are used in this manuscript:

AHI	Apnea-Hypopnea Index
BMI	Body mass index
CD	Circulatory delay
CI	Confidence interval
CSR	Cheyne-Stokes respiration
EF	Ejection fraction
HF	Heart failure
HFrEF	Heart failure with reduced ejection fraction
HSAT	Home sleep apnea test
IQR	interquartile range
LECT	Lung-to-ear circulation time
LFCT	Lung to finger circulation time
OSA	Obstructive sleep apnea
PSG	Polysomnogram
SDB	Sleep-disordered breathing
Sec	seconds
Tcirc	Circulation time
TTE	Transthoracic echocardiogram
T88	Time in minutes with oxygen saturation <88%
